# Elevation Determines Fungal Diversity, and Land Use Governs Community Composition: A Dual Perspective from Gaoligong Mountains

**DOI:** 10.3390/microorganisms12112378

**Published:** 2024-11-20

**Authors:** Zhuanfei Zeng, Ruilong Huang, Wei Li

**Affiliations:** College of Soil and Water Conservation, Southwest Forestry University, Kunming 650224, China; zzf2002428@163.com (Z.Z.); hrl13413153274@163.com (R.H.)

**Keywords:** soil fungal community, land-use practices, elevation, high-throughput sequencing, Gaoligong mountain

## Abstract

Soil fungi are closely tied to their surrounding environment. While numerous studies have reported the effects of land-use practices or elevations on soil fungi, our understanding of how their community structure and diversity vary with elevation across different land-use practices remains limited. In the present study, by collecting soil samples from four different land uses in the Gaoligong Mountain area, namely shrublands (SLs), coffee plantations (CPs), cornfields (CFs), and citrus orchards (COs), and combining them with the changes in altitude gradients (low: 900 m, medium: 1200 m, high: 1500 m), high-throughput sequencing technology was used to analyze the composition and diversity of soil fungal communities based on the collected soil samples. The results showed that the interaction between land-use types and elevation significantly influenced the structure and diversity of fungal communities, although their relative importance in shaping fungal diversity or community structure varied. Specifically, elevation posed a stronger effect on fungal community alpha-diversity and functional guilds, whereas land-use types had a greater influence over fungal community composition. Our study reveals the individual and combined effects of land-use practices and elevation on the structure and diversity of soil fungal communities in the Gaoligong Mountain region, enhancing our understanding of the distribution patterns and driving mechanisms of soil fungal communities in this biodiversity-rich region.

## 1. Introduction

Soil microorganisms play essential ecological roles in terrestrial ecosystems. They facilitate nutrient exchange between vegetation and soil, regulate biogeochemical cycles and litter decomposition, sustain agricultural development, and provide valuable ecological services to human society [1,2]. In recent years, the rapid population growth and continuous expansion of the human footprint have led to extensive and profound impacts on the structure, processes, and functions of global ecosystems through changes in land-use practices [3]. Soil under native vegetation harbors rich and diverse microbial communities [4]. However, disturbances from human activities, such as agriculture, grazing, urbanization, and industrialization, disrupt the integrity of native vegetation and often cause significant changes in soil microbial structure, diversity, and functioning, further harming soil stability and health [5,6]. Moreover, soil microbial diversity varies significantly across different land-use types [7]. For instance, land-use types, such as cropland, grazed grassland, and plantations, significantly alter species composition and distribution of soil microorganisms, thereby affecting biogeochemical processes and soil fertility [8]. Also, certain land-use practices can enhance microbial community diversity, while others may be detrimental to microbial survival [9]. Differences in soil microbial diversity not only profoundly affect soil ecosystem functioning but also directly impact the provision of crucial ecosystem services on which human society heavily relies [10]. Therefore, in-depth research on the factors influencing soil microbial communities and their ecological functions is crucial for achieving a harmonious coexistence between humans and nature.

In mountainous ecosystems, specific fungi (such as saprophytic fungi, mycorrhizal fungi, etc.) play a crucial role in regulating nutrient cycling [11], organic matter decomposition [12], plant growth, and ecosystem stability [13,14,15]. In addition, many fungi form symbiotic relationships with plants to support their growth and stress resistance, especially in high-altitude areas with low soil fertility and high environmental pressure. However, with the transformation of land use in mountainous ecosystems, the structure and function of soil fungal communities also change accordingly. For example, converting primary rainforests into pastures can lead to a dramatic decrease in soil fungi diversity, particularly affecting the proportion of saprophytic fungi and ectomycorrhizal fungi [16,17]. This also significantly reduces the role of fungal communities in soil. Even in highly anthropogenically influenced land types such as grazed land, mowed land, and farmland, there are significant differences in the diversity levels of fungal communities [18]. Altitude also influences the structure and diversity of fungal communities [19]. Elevation gradients are often associated with significant climate changes, such as temperature decreases and changes in precipitation patterns, which can affect vegetation types and distribution as key climate drivers. For example, higher temperatures in low-altitude areas are conducive to the cultivation of certain economic crops, while cold-resistant shrubs and local vegetation in high-altitude areas are better suited for growth. Changes in vegetation types can indirectly affect the structure and diversity of microbial communities by altering soil organic matter and litter characteristics. In other words, the effect of altitude on microbial communities is mediated through vegetation succession driven by climate [20]. In addition, factors such as soil physical and chemical properties, which can vary with increasing altitude, may affect fungal community diversity [21]. Some studies have found that increasing altitude may increase soil acidity or reduce soil nutrient content, which in turn reduces fungal diversity [22]. Other studies have found that fungal diversity may increase with altitude, possibly due to enhanced niche differentiation among fungi species at higher elevations [23]. Furthermore, altitude affects the distribution patterns of fungal communities, resulting in significant differences in the composition and abundance of fungi at various altitudinal levels [24]. For instance, many studies have demonstrated that both the abundance and richness of arbuscular mycorrhizal fungi (AMF) and ectomycorrhizal fungi decrease with increasing altitude [25,26,27]. On Mila Mountain in the Qinghai-Tibet Plateau, AMF richness decreased with increasing altitude on the eastern slope but showed a unimodal pattern on the western slope [28]. Some studies also reported that fungal abundance and diversity exhibited a unimodal pattern with altitude, peaking at mid-altitudes [29]. Additionally, other studies have observed a monotonic decrease [30], monotonic increase [31], hollow distribution pattern [32], or no relationship at all [33,34] between altitude and fungal diversity. Overall, in contrast to the more consistent monotonic decrease or unimodal relationships typically observed between plant and animal diversity and altitude [35], fungal community diversity exhibits more varied and complex patterns with altitude. This complexity may arise from the interplay of various factors, including geographical location, vegetation type, climate, soil properties, and human activities. Research in this area enhances our understanding of the mechanisms that shape and maintain soil fungal diversity. However, current research often focuses on the relationship between individual factors and fungal community characteristics, frequently neglecting the combined or comparative effects of these factors. For example, while many studies have tested the effects of land use or altitude on soil fungal communities, research on the combined and comparative effects of both factors on fungal communities remains limited. In addition, studying fungal communities in mountain ecosystems not only helps to understand the operational mechanisms of mountain ecosystems, but also provides theoretical support for the protection and restoration of mountain environments. Therefore, focusing on the diversity and ecological functions of fungal communities is crucial for exploring the health and sustainability of mountain ecosystems.

The Gaoligong Mountains, situated in the southwestern frontier of China, represent a convergence zone for three global biodiversity hotspots, namely, the Himalayas, the Indo-Burma region, and the mountains of southwestern China [36]. Its unique geographical location, complex terrain, and diverse climate have created a unique ecological environment. The interconnected ecosystems under different altitudes and microclimate conditions make it an ideal place for studying ecological adaptation and the evolution of species diversity. Like other global biodiversity hotspots, however, the biodiversity of the Gaoligong Mountains faces severe threats from human activities. In recent years, Gaoligong Mountain and its surrounding areas have faced increasingly severe environmental pressure. Human activities such as agricultural expansion, deforestation, and road construction have destroyed the original vegetation in the area, leading to the degradation of local ecosystems. These environmental challenges not only affect biodiversity but also alter soil structure and nutrient cycling, significantly impacting the composition and function of soil fungal communities. Local residents rely heavily on cultivating crops such as coffee and corn for their economic livelihood, frequently adjusting their crop choices in response to market demands. Although some studies have begun to investigate the effects of land use on vascular plants and vertebrates in the Gaoligong Mountain region [37,38], research on how land use affects soil fungal community structure and diversity through altering soil physicochemical properties remains limited. Therefore, studying the structure and function of fungal communities is very suitable in this unique environment. It can not only provide data support for understanding the ecological characteristics of Gaoligong Mountain but also helps to develop scientific conservation strategies.

The present study aims to investigate changes in species composition and diversity of soil fungal communities along an altitudinal gradient across four typical land-use types in the Gaoligong Mountain region, including shrubland (SL), coffee plantations (CPs), cornfields (CFs), and citrus orchards (COs). We aim to address the following questions: (1) Are there significant differences in species composition and diversity of fungal communities across different land-use types? (2) Are there significant differences in species composition and diversity of fungal communities at varying altitudes? (3) Do land-use types and altitude jointly influence the species composition and diversity levels of fungal communities? (4) Is the impact of land use on species composition and diversity of fungal communities more significant than the effect of altitude, or vice versa?

## 2. Materials and Methods

### 2.1. Study Area

The Gaoligong Mountains (98°55′–99°10′ E, 25°36′–28°57′ N) are located in the Nujiang autonomous prefecture and the western part of Baoshan City, Yunnan Province, southwest China. This region experiences a subtropical plateau monsoon climate, with an average annual temperature ranging from 14 to 15 °C. Influenced by the southwest monsoon winds from the Indian Ocean, the western slopes receive significantly more precipitation than the eastern slopes, and the peaks of the mountains can receive up to 3600 mm of precipitation [39]. The main vegetation type is the western slopes receive significantly more precipitation than the eastern slopes, and the predominant soil types are yellow-red soil, brown-red soil, gray-calcareous soil, and alpine meadow soil [40]. This study was conducted mainly in Baihualing village, situated on the eastern slope of the southern section of the Gaoligong Mountains, within the territory of Mangkuan Town, Longyang District, Baoshan City, Yunnan Province. The village is under the jurisdiction of the Baoshan Management Bureau of the Gaoligong Mountains National Nature Reserve.

### 2.2. Sample Collection

**Figure 1:**
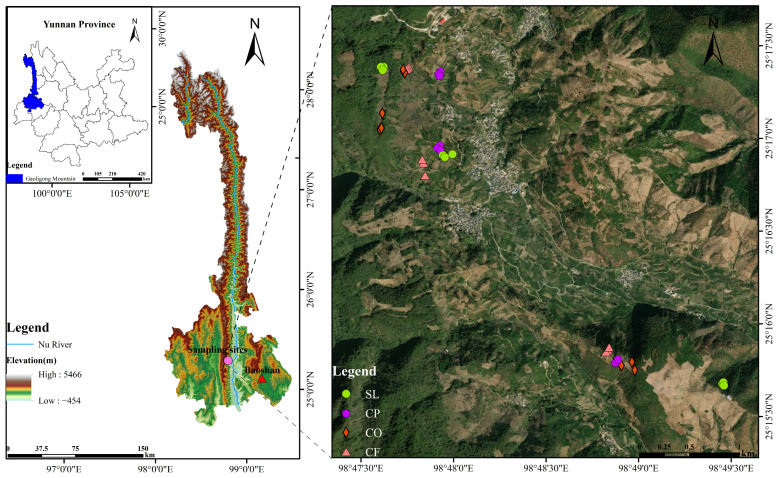
Study area and sampling site.

Soil samples were collected in January 2024. The sampling site is located in Baihualing village on the eastern slope of the southern section of the Gaoligong Mountain, and four representative local land uses were selected, namely shrubland (SL), coffee plantations (CPs), cornfields (CFs), and citrus orchards (COs). An elevation interval of 300 m was set, and three elevation intervals along low: 900–1200 m, middle: 1200–1500 m, and high: 1500–1800m (uniformly use 900 m, 1200 m, and 1500 m to indicate the low, medium, and high altitude in the following). Three replicate sample plots were set up in each habitat for a total of 36 soil samples, and a map of specific sampling points is shown in Figure 1. Visible plant debris from around the soil was removed and a sterile spade was used to randomly select five points along the “S” curve and collect surface soil (0–20 cm) when sampled. Using sterile gloves to remove plant roots, gravel, and other debris, after being sieved through 2 mm mesh, part of the soil samples was stored in a dry room temperature environment for the analysis of soil physicochemical properties, and the other part was stored in a dry ice incubator at −80 °C for the testing of soil microorganisms.

### 2.3. Measurements of Soil Physicochemical Properties

Soil pH was measured using a portable pH meter (PHS-3E). Soil organic carbon (SOC) and total carbon (TC) were measured by an elemental analyzer, total nitrogen (TN) content was measured by the Kjeldahl digestion method (Kjeldahl nitrogen meter, Haineng K9840, Shandong Haineng Scientific Instrument Co., Ltd., Dezhou, Shandong, China), and total phosphorus (TP) content was measured by the molybdenum antimony colorimetric method. Ammonium nitrogen (NH_4_^+^-N) content was determined by LY/T1228-2015 Indophenol Blue colorimetry, nitrate nitrogen (NO_3_^−^-N) content was determined by UV-Vis spectrophotometer, dissolved organic carbon (DOC) was determined by the water leaching-potassium dichromate oxidization method, and soil water content (SWC) was determined by the drying method [41].

### 2.4. Soil DNA Extraction and ITS rRNA Sequencing

Total soil DNA was extracted from fresh soil samples following the manufacturer’s instructions. Genomic DNA integrity was detected by agarose gel electrophoresis, while Nanodrop 2000 (Thermo Fisher Scientific, Waltham, MA, USA) was used to detect genomic DNA quality. The primers ITS1 (5′-CTTGGTCATTTAGAGGAAGTAA-3′) and ITS2 (5′-GCTGCGTTCTTCATCGATGC-3′) were used for amplification of the fungi. PCR reactions were performed in a 25 μL mixture, and used the PCR reaction conditions reported by Sui et al. [42]. Agarose gel electrophoresis was used to detect whether the amplification products were single and specific. Three parallel amplification products from the same sample were mixed, and the products were purified by adding an equal volume of AgencourtAMPureXPPCR Purification Beads to each sample. Finally, paired-end sequencing was performed on the Illumina MiSeq platform (2 × 250 bp). Raw sequencing data obtained from the Illumina MiSeq platform were processed using QIIME2 to ensure reproducibility. Species annotation of ASV sequences was performed with a confidence threshold of 0.6 for ITS regions, using the default classify-sklearn method. The ASV analysis in this study was conducted at the species level. Sequencing reads were quality-filtered, demultiplexed, and merged to generate representative sequences, with chimeric sequences removed using the DADA2 plugin in QIIME2. The resulting high-quality reads were clustered into amplicon sequence variants (ASVs) for finer taxonomic resolution, and taxonomic classification was performed using the UNITE database (version 9.0) for fungal ITS sequences. Rarefaction curves were computed and plotted using R software (version 4.3.3). The rarefaction curve tended to flatten (Figure S1), indicating that the sequencing depth is sufficient to reflect the actual soil microbial community.

### 2.5. Data Analyses

A two-way analysis of variance (ANOVA) and Duncan’s test were performed on the differences in soil physicochemical properties, fungal community composition, and diversity under different land-use patterns and elevations using SPSS 27.0, and Spearman’s rank correlation analysis was used to explore the correlation between the relative abundance of fungal community composition, fungal alpha diversity, fungal functional guilds, and soil physicochemical properties. Principal coordinate analysis (PCoA) was finished using the R software (version 4.3.3) “Vegan” package based on Bray–Curtis distances to analyze the similarity of fungal communities between land uses and altitudes. A permutation-multivariance analysis of variance (PERMANOVA) was then used to test for differences in soil fungal communities between the two. Redundancy analysis (RDA) was performed using Canoco5 software to investigate the association between soil physicochemical properties and fungal community composition. The FunGuild database was used to classify fungal ASVs by function to predict their ecological roles [43]. According to the classification results from FunGuild, the reliability level of the functional annotation is “probable”, indicating that the confidence in the identified functions is at a moderate level. Finally, plots were made using Origin 2022 and GraphPad Prism 9.5.1.

## 3. Results

### 3.1. Soil Physicochemical Properties

**Figure 2:**
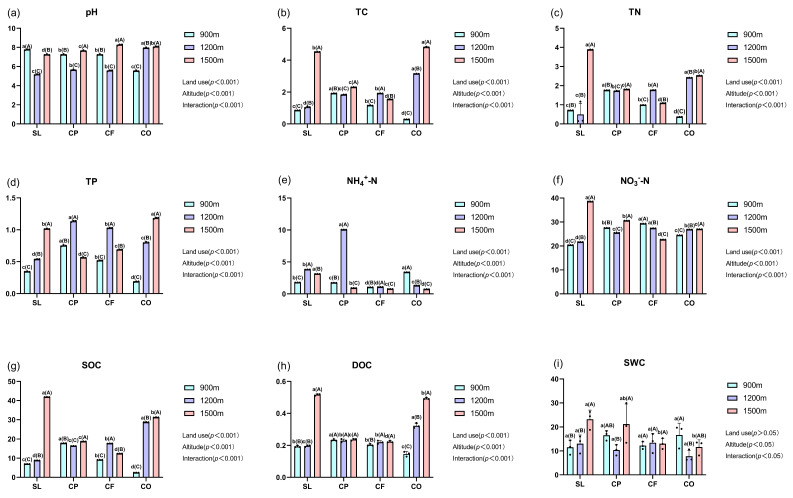
Soil physicochemical properties with different land uses and altitudes in the Gaoligong Mountains. (Different letters indicate significant levels (*p* < 0.05). At the same elevation, significant differences between different land uses are denoted by lowercase letters (e.g., a); at the same land use, significant differences between different elevations are denoted by uppercase letters (e.g., A). (**a**): pH. (**b**): TC, total carbon. (**c**): TN, total nitrogen. (**d**): TP, total phosphorus. (**e**): NH_4_^+^-N, ammonium-nitrogen. (**f**): NO_3_^−^-N, nitrate-nitrogen. (**g**): SOC, soil organic carbon. (**h**): DOC, dissolved organic carbon. (**i**): SWC, soil water content).

Except for SWC, other soil physicochemical properties were significantly different among the land uses. At 900 m, shrubland (SL) had the highest content of pH, and coffee plantations (CPs) had a higher content of TC, TN, TP, SOC, and DOC than the other three land uses. At 1200 m, citrus orchards (COs) had the highest levels of pH, TC, TN, SOC, and DOC. At 1500 m, cornfields (CFs) had the highest pH and the lowest levels of TC, TN, NO_3_^−^-N, SOC, and DOC, and shrubland (SL) had the physicochemical properties of TN, NH_4_^+^-N, NO_3_^−^-N, SOC, and DOC over the other land uses (Figure 2).

The change in elevation caused significant changes in the physicochemical properties of soil (Figure 2). Including TC, TP, NO_3_^−^-N, SOC, DOC, and SWC in shrubland (SL), soil physicochemical properties showed an increasing trend with elevation, while in coffee plantations (CPs), there was not upward or downward trend with elevation, and the physicochemical properties reached a maximum at 1500 m, except for TP and NH_4_^+^-N. In cornfields (CFs), NO_3_^−^-N content showed a decreasing trend with elevation, while in the citrus orchards (COs), pH, TC, TN, TP, NO_3_^−^-N, SOC, and DOC showed an increasing trend with elevation, and the value of NH_4_^+^-N showed a decreasing trend with elevation.

### 3.2. Composition of Soil Fungal Community

In all soil samples analyzed, a total of 3,075,190 raw sequences were obtained using the Illumina NovaSeq sequencing platform (Illumina, Inc., San Diego, CA, USA). After quality filtering, 2,721,888 sequences remained, and denoising yielded 2,680,023 sequences. Subsequent merging resulted in 2,394,385 sequences, and finally, 2,389,152 high-quality sequences were obtained after additional processing steps. Among these, 602,819, 591,388, 584,041, and 610,904 sequences were derived from shrubland, coffee plantations, cornfields, and citrus orchards, respectively, while 786,225, 791,310, and 811,617 sequences were obtained from low, middle, and high altitudes, respectively.

**Figure 3:**
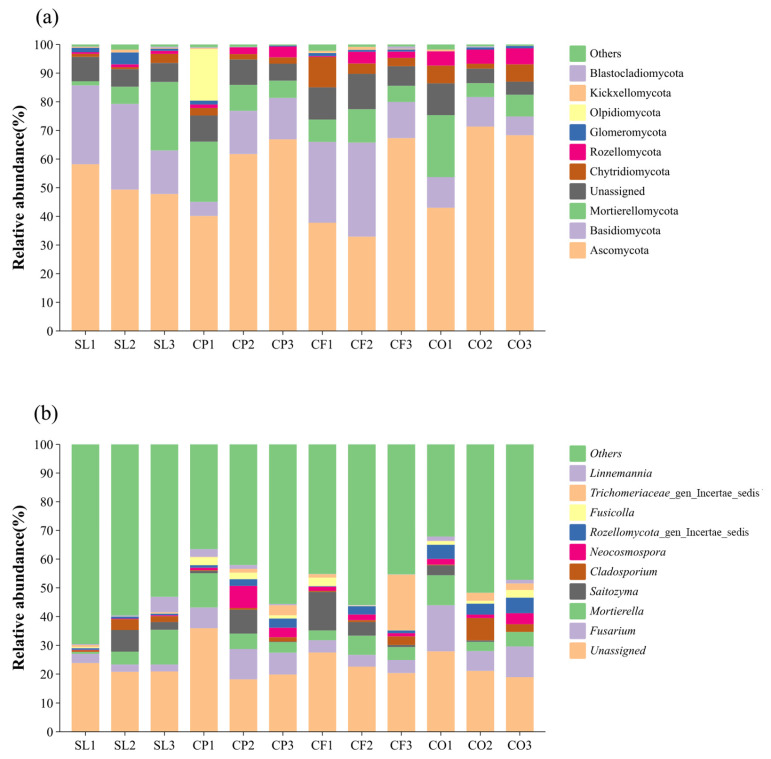
Relative abundance of soil fungal phyla (**a**) and fungal genera (**b**). (SL1, SL2, and SL3 correspond to low, medium, and high elevation shrublands, respectively, and the same applies to other land uses. SL: shrubland, CP: coffee plantation, CF: cornfield, CO: citrus orchard).

In the low-altitude region, a total of 5192 ASVs were identified, distributed across 15 phyla, 59 classes, and 643 genera. In the middle-altitude region, 5180 ASVs were identified, spanning 16 phyla, 56 classes, and 743 genera. In the high-altitude region, 5422 ASVs were identified, distributed among 17 phyla, 59 classes, and 779 genera. The dominant phyla across all altitudes were Ascomycota and Basidiomycota, with an average relative abundance of more than 10%. Ascomycota had the highest relative abundance, with a relative abundance higher than 60% in CP2, CP3, CO2, CO3, and CF3, with the highest abundance in CO2 at 71.34%. The relative abundance of Basidiomycota was the highest in SL and CF, and reached the maximum in CF2, with a relative abundance of 32.84%. Besides, the average abundance of Mortierellomycota in CP reached 12.02%, which was higher than the other three land uses. In SL, the relative abundance of Ascomycota decreased with increasing elevation, while the opposite was observed in CP. The relative abundance of Mortierellomycota increased with increasing elevation in SL, while the opposite was observed in CP, the relative abundance of Chytridiomycota decreased with increasing elevation in CF. It’s worth noting that the relative abundance of Olpidiomycota reached 18.03% in CP1, which was much higher than the other sample sites. The presence of unclassified fungal phyla was also high (Figure 3).

*Fusarium* and *Mortierella* had the highest relative abundance in all soil samples, with an average relative abundance greater than 5%. *Fusarium* (16.04%) had the highest percentage in CO1, and the relative abundance of *Mortierella* (12.06%) in SL3 exceeded that of the other samples. Similarly, the relative abundance of *Mortierella* increased with elevation in SL and decreased with elevation in CP. From Table 1, it can be seen that, except for Blastocladiomycota, the effect of land use on each fungal phylum was the most significant, and the interaction between land use and elevation had a significant effect on the abundance of other soil fungal phyla.

### 3.3. Soil Fungal Community Diversity

**Figure 4:**
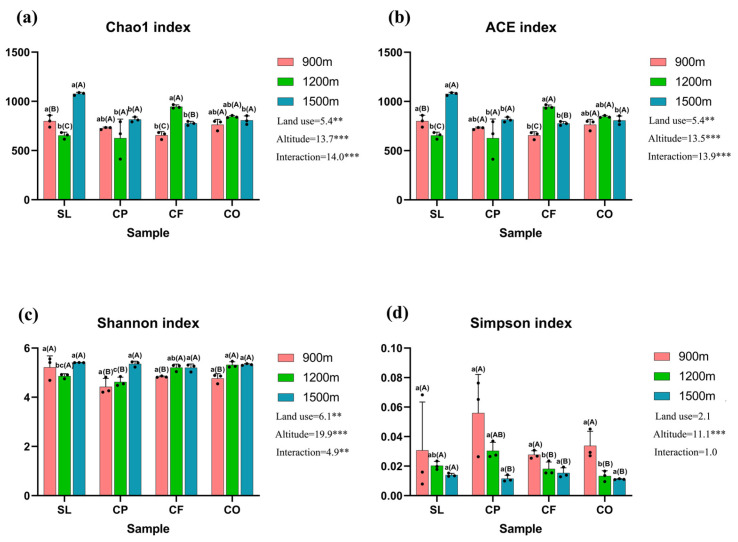
Alpha diversity of soil fungal community. ((**a**): Chao1 index; (**b**): ACE index; (**c**): Shannon index; (**d**): Simpson index). Lowercase letters indicate significant differences in alpha diversity of fungal communities between different land uses (*p* < 0.05); uppercase letters indicate significant differences between different elevations (*p* < 0.05). The values on the right side of each graph represent the F-value and significance results. ** *p* < 0.01; *** *p* < 0.001.

Land use, elevation, and the interaction between the two had significant impacts on the Chao1 index, the ACE index, and the Shannon index (*p* < 0.01), but land use and interaction did not have a significant impact on the Simpson index (*p* > 0.05) (Figure 4).

There were no significant differences in the Shannon and Simpson indexes among land uses at 900 m and 1500 m elevation, while at 1200 m elevation, there were significant differences in the Shannon and Simpson indexes for CP and CO (*p* < 0.05). The richness indices of SL and CP decreased and then increased with elevation, while CF and CO showed the opposite pattern of increasing and then decreasing. Except for SL, the Shannon index of the other three land uses basically increased with elevation.

**Figure 5:**
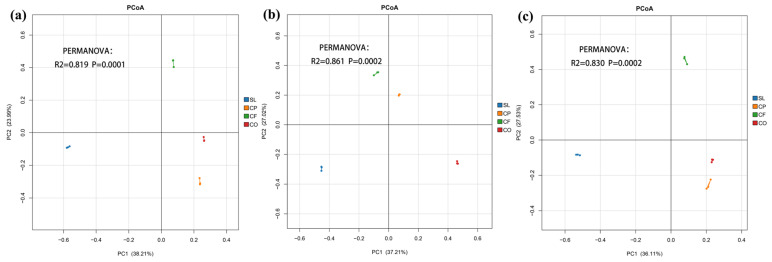
PCoA analyses of the soil fungal community. ((**a**–**c**) correspond to 900 m, 1200 m and 1500 m).

PCoA analysis of soil fungal community structure based on the Bray–Curtis distance was performed to assess the similarities and differences in the results of different fungal community compositions in the Gaoligong Mountains, and the amount of PC interpretation at each altitude level is shown in Figure 5. There was a significant separation distance between the different land uses, which, combined with the results of the PERMANOVA analysis, suggests that the different land uses resulted in extremely significant differences in fungal community composition (*p* < 0.001); in particular, the shrubland was in the third quadrant, with a greater distance from the other three land uses, suggesting that there was a markedly different pattern of community structure between the shrubland and the other three sample sites. The change in elevation caused the position of the CP to change back and forth between the first and fourth quadrants, while the location of the other sample plots was shifted very little, so the effect of altitude alone on CP was more significant. In summary, the effect of land use on fungal community composition was greater than elevation.

### 3.4. Functional Guild of Soil Fungal Community

**Figure 6:**
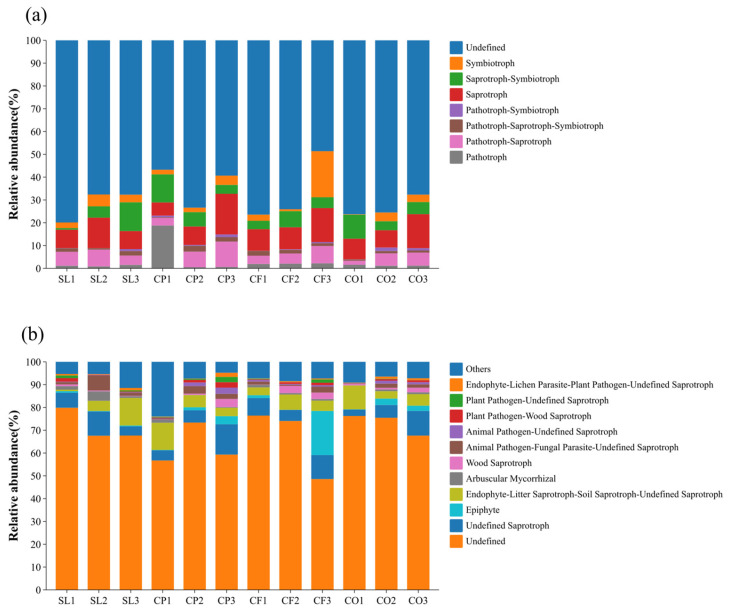
Relative abundance of fungal trophic mode (**a**) and guild (**b**). SL1, SL2, and SL3 correspond to low, medium, and high-elevation shrublands, respectively, and the same applies to other land uses. SL: shrubland, CP: coffee plantation, CF: cornfield, CO: citrus orchard.

When determining functional guilds, only select those guilds with relative abundance higher than 1% for analysis. Considering the three altitude gradients in this study, we standardized these functional guilds by only including functional guilds that consistently detected sufficient abundance at all altitudes to maintain comparability across the three altitude gradients. In addition, trophic mode is a broad classification of fungal nutritional patterns, while guilds further subdivide these patterns, reflecting the specific functions performed by fungi in ecosystems (Figure 6). Based on the FUNGuild database, the major functional guilds in the soil fungal communities of the sampled sites were Pathotroph, Pathotroph-Saprotroph, Pathotroph-Saprotroph-Symbiotroph, Pathotroph-Symbiotroph, Saprotroph, Saprotroph-Symbiotroph, and Symbiotroph. The dominant trophic mode by mean relative abundance was highest in Saprotroph in all sample plots, the highest in SL3 and CO1 was Saprotroph-Symbiotroph (12.62%, 10.50%), Pathotroph was the highest in CP1 with 18.78% and Symbiotroph (20.20%) was the dominant trophic mode in CF3. Saprotroph was dominant in most of the samples with relative abundance ranging from 7.57% to 17.82% (Figure 6).

The functional guild measured in each sample continued to be classified by average relative abundance levels above 1%, yielding 10 functional fungi, as shown in Figure 6. Among them, Undefined Saprotroph and Endophyte-Litter Saprotroph-Soil Saprotroph-Undefined Saprotroph were the dominant functional fungi. It is noteworthy that Epiphyte had the highest percentage of 19.35% among CF3, which was significantly higher than the other samples.

The results in Table 2 reflect that land use did not have a significant effect on the relative abundance of Undefined Saprotroph and did not catch up with elevation significantly for some other functional fungi. However, the interaction between the two had a very significant effect on the relative abundance of functional guilds of fungi.

### 3.5. Relationship Between Soil Fungal Community and Environmental Factors

#### 3.5.1. Relationship Between Fungal Community Composition and Environmental Factors

**Figure 7:**
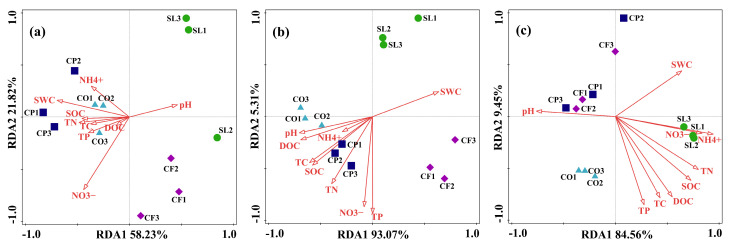
RDA analysis applied to soil fungal community data and soil physicochemical properties. ((**a**–**c**) correspond to 900 m, 1200 m and 1500 m).

In this study, in order to analyze how land use and altitude regulate fungal community structure through their impact on soil physicochemical properties, we used environmental variables (such as pH, TC, TN, NH_4_^+^-N, NO_3_^−^-N, etc.) as independent variables, and the relative abundance of the top 10 phyla as dependent variables. All soil variables were standardized prior to analysis to ensure consistency across different variable scales. As shown in Figure 7a, the explanation rates of the first and second axes were 58.23% and 21.82%, respectively, where SWC (*p* < 0.05), NH_4_^+^-N (*p* < 0.01), and NO_3_^−^-N (*p* < 0.05) were the key environmental factors influencing the composition of the fungal community at low altitude, with the explanation rates of 28.9%, 19.6%, and 18%, respectively. At medium altitude, pH (*p* < 0.05) and NH_4_^+^-N (*p* < 0.01) had a greater effect on fungal community composition, with explanation rates of 46.6% and 35.5%, respectively. At high altitude, NH_4_^+^-N (*p* < 0.01) was the key environmental factor affecting fungal community composition with an explanation rate of 75.6%.

**Figure 8:**
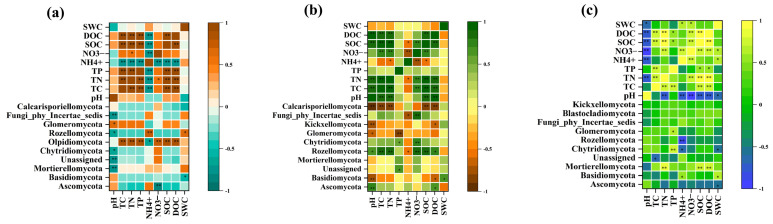
Correlation between the 10 most abundant fungal phyla and the physicochemical properties. ((**a**–**c**) correspond to 900 m, 1200 m and 1500 m). * *p* < 0.05; ** *p* < 0.01.

A heat map analysis of the correlation between the top ten fungal phyla in terms of relative abundance and physicochemical properties (Figure 8) showed that pH was extremely significantly negatively correlated with Mortierellomycota, Unassigned, and Fungi_phy_Incertae_sedis, significantly negatively correlated with Chytridiomycota and Rozellomycota, and significantly positively correlated with Glomeromycota at low altitudes. Olpidiomycota was extremely significantly positively correlated with TC, TN, TP, NO_3_^−^-N, SOC, and DOC. In addition, at mid-altitude, pH extremely significantly affected Ascomycota and was extremely significantly negatively correlated with Basidiomycota, Kickxellomycota, and Calcarisporiellomycota. Rozellomycota was affected by most of the physicochemical factors which extremely significantly positively correlated with TC, TN, NO_3_^−^-N, and SOC, then a significant positive correlation with pH and DOC and a significant negative correlation with NH_4_^+^-N. Finally, at high altitude, pH had no significant effect on the fungal phylum, and NH_4_^+^-N was significantly positively correlated with Basidiomycota, significantly negatively correlated with Chytridiomycota, and extremely significantly negatively correlated with Rozellomycota.

#### 3.5.2. Relationship Between Fungal Community Diversity and Environmental Factors

Spearman’s correlation between fungal community diversity and physicochemical properties is shown in Table 3. It can be seen that NH_4_^+^-N and NO_3_^−^-N are the key factors affecting the diversity of soil fungal community. pH only has a highly significant negative correlation with the diversity of fungal communities at high altitude.

#### 3.5.3. Correlation Between Functional Fungal Guilds and Environmental Factors

**Figure 9:**
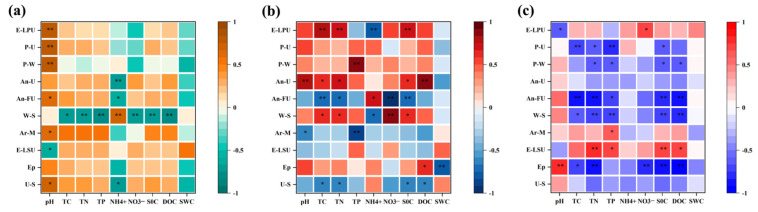
Heat map of the correlation between functional guild of fungi and physicochemical properties. ((**a**–**c**) correspond to 900 m, 1200 m and 1500 m). * *p* < 0.05, ** *p* < 0.01. U-S: Undefined Saprotroph, Ep: Epiphyte, E-LSU: Endophyte-Litter Saprotroph-Soil Saprotroph-Undefined Saprotroph, Ar-M: Arbuscular Mycorrhizal, W-S: Wood Saprotroph, An-FU: Animal Pathogen-Fungal Parasite-Undefined Saprotroph, An-U: Animal Pathogen-Undefined Saprotroph, P-W: Plant Pathogen-Wood Saprotroph, P-U: Plant Pathogen-Undefined Saprotroph, E-LPU: Endophyte-Lichen Parasite-Plant Pathogen-Undefined Saprotroph.

Spearman’s correlation of fungal functional guilds with physicochemical factors is shown in Figure 9. At low altitude, pH and NH_4_^+^-N were the key environmental factors affecting functional fungi. pH was significantly positively correlated with Undefined Saprotroph, Arbuscular Mycorrhizal, and Animal Pathogen-Fungal Parasite-Undefined Saprotroph, and showed extremely significant positive correlation with Plant Pathogen-Wood Saprotroph, Plant Pathogen-Undefined Saprotroph, and Endophyte-Lichen Parasite-Plant Pathogen-Undefined Saprotroph. Wood Saprotroph is a functional fungus with the most pronounced effect of physicochemical factors. TC, TN, and SOC were the main factors affecting functional fungi at middle altitude, and Undefined Saprotroph was significantly negatively correlated with TC, TP, SOC, and DOC. From Figure 9c, physicochemical factors have a great influence on functional fungi at high altitude. The effect of physicochemical factors on the functional fungal guilds became more and more significant with the increase in altitude.

## 4. Discussion

The present study explores the effects of land-use types, altitude, and their interactions on the structure and diversity of soil fungal communities in the Gaoligong Mountain region. It also examines the potential mechanisms driving changes in different microbial communities by analyzing the variation in soil physicochemical properties.

### 4.1. The Impact of Land Use on Soil Physicochemical Properties and Fungal Communities

Economic and social development has led to changes in land-use patterns worldwide, imperiling soil biodiversity and compromising ecosystem functions and services. These changes are particularly pronounced in mountain ecosystems [44]. In the Gaoligong Mountain region, local residents have gained economic benefits from cultivating cash crops such as coffee. However, this shift in land use has created an imbalance between conservation and development [45]. Changes in land use can alter the physicochemical properties and structure of the soil, resulting in variations in the structure and diversity of microbial communities [46]. According to our results, land-use practices significantly affected various soil physicochemical properties (except for SWC). For example, in low-altitude regions, the TN and SOC content in coffee plantations (CPs) were significantly higher compared to soils under the other three land-use types (Figure 2). Similarly, at mid-altitudes, the TN and SOC content in citrus orchards (COs) were significantly higher than in soils under the other land-use types. Soil nutrient indicators, such as TN and the carbon-to-nitrogen ratio, are often closely related to soil organic carbon content. Studies have shown a strong positive correlation between TN and soil organic carbon content [47], a pattern that is also supported by our findings (Figure 8). The relatively warmer climate at low altitudes may promote plant growth and organic matter accumulation [48]. Coffee thrives well in these conditions, and the increased plant residues contribute to higher SOC [49]. At mid-altitudes where temperatures are lower, citrus may be more adaptable than coffee, producing more organic materials that decompose more slowly, thereby increasing SOC content. Additionally, different agricultural management practices may also contribute to these differences. Traditional coffee plantations often rely heavily on chemical fertilizers and pesticides to boost yields, with less emphasis on the return of organic matter to the soil. This approach can lead to nutrient loss and reduced organic matter accumulation and SOC [50,51]. Although the warm climate at lower altitudes may favor coffee growth and organic matter accumulation, traditional farming methods that rely heavily on chemical fertilizers can inhibit long-term SOC accumulation. In contrast, organic citrus orchards, which minimize the use of chemical fertilizers and increase the application of organic materials (e.g., compost and green manure), tend to promote greater SOC accumulation at mid-altitudes [52]. Thus, the higher SOC content in mid-altitude citrus orchards may be related to the improvement in soil health resulting from organic farming practices. In contrast, traditional coffee plantations often rely more on external chemical inputs and lack organic matter replenishment. Although low-altitude coffee plantations have relatively high soil organic carbon (SOC) content, this accumulation may be more attributable to the natural environment rather than the long-term effects of cultivation practices. We also found that SWC was not significantly affected by land-use changes. However, previous studies have shown that SWC is significantly influenced by land-use types [53,54], especially through the complex effects of vegetation type on soil moisture [55]. Since SWC is influenced by multiple factors, such as climate, land-use changes, altitude, and soil depth [56], in the present study, altitude might have played a leading role in determining SWC, masking the effects of land-use practices. Variations in altitude typically lead to changes in temperature, humidity, and precipitation, which often have a greater impact on SWC than land use. Additionally, in the Gaoligong Mountain region, most precipitation occurs during the rainy season [57], while soil moisture replenishment is inadequate during the dry season. This seasonal precipitation pattern may result in unstable soil moisture levels, making it difficult to alleviate low moisture conditions during the dry season, even in the presence of varying land-use practices.

Different land-use types significantly affect the composition of microbial communities. In our study, land-use-induced differences in soil physicochemical properties seem to play a key role in the observed changes in fungal communities. Regardless of land-use types, the dominant phyla in the soil samples are Ascomycota and Basidiomycota (Figure 3), while the dominant genera are *Fusarium* and *Mortierella*. The average relative abundance of Ascomycota was highest in CO, especially at mid-altitudes, where it reached its peak (Figure 3a). We also found that in mid-altitude citrus orchards, soil pH, TC, TN, SOC, and DOC levels were higher compared to other land-use types, suggesting that favorable pH and nutrient conditions promote the growth and reproduction of Ascomycota, aligning with previous studies [58]. In contrast, the average relative abundance of Ascomycota was lowest in CF, likely due to the significant influence of agricultural activities such as fertilizer application [59]. Notably, in low-altitude coffee plantations (CP1), the relative abundance of Olpidiomycota was significantly higher than in other groups (Figure 3), and the levels of TC, TN, TP, SOC, and DOC were also the highest. The results of Spearman’s analysis showed a significant positive correlation between the relative abundance of Olpidiomycota and these physicochemical properties, indicating that higher soil fertility levels have a clear promoting effect on the abundance of Olpidiomycota [60]. Our results also revealed that land use significantly affected the Shannon index of soil fungi, but not the Simpson index., which is consistent with the recent study reported by Kerfahi et al. [61,62]. Our results also suggest that fungal community diversity is influenced by TN, NH_4_^+^-N, NO_3_^−^-N, and SOC, which is consistent with other research findings [30]. Among the four land-use types, fungal diversity was highest in SL, as indicated by the average values of the Chao1 index, the ACE index, and the Shannon index across different altitudes (Figure 4). Unlike the other three land-use types, shrubland is less affected by human activities in general, and by agricultural management in particular, which may offer more stable habitat conditions for fungi. In contrast, more intensive agricultural management might have disrupted soil stability and inhibited fungal community diversity. Overall, our findings align with other studies, which have shown that fungal diversity is significantly lower in typical agricultural lands compared to natural forests [63,64]. However, soil fungal diversity is not influenced solely by soil physicochemical properties, as it can also be affected by human activities, geographic location, and seasonal changes [65]. Our findings showed that among all land-use types, the most dominant functional fungal guild (based on average relative abundance) was Saprotroph (Figure 6a), and the dominant functional fungus was also saprophytic. The shift in the dominance of Saprotroph can significantly affect soil fertility and carbon dynamics. For example, an increase in saprophytic fungi can enhance the decomposition of litter and contribute to the mineralization of organic matter, providing carbon and energy sources for plant growth. This process not only improves soil fertility but also promotes carbon dynamics by converting organic carbon into a form that is more easily absorbed by plants [66]. Numerous studies have shown that arbuscular mycorrhizal fungi form symbiotic relationships with more than 90% of plant species [67]. In particular, the relationship between AMF and citrus can enhance fruit growth and increase yield [68]. However, in our study, the relative abundance of AMF in CO was the lowest (Figure 6b). One possible explanation is that the high pH and TP content in citrus orchard soils might have posed a detrimental impact on AMF [69,70,71].

### 4.2. The Impact of Altitude on Soil Physicochemical Properties and Fungal Communities

This study reveals significant differences in soil physicochemical properties across altitudes ranging from 900 to 1500 m (Figure 2). For example, the pH in CO showed an increasing trend with altitude, and the vegetation coverage and litter accumulation in high-altitude regions differ from those at lower altitudes. Lower temperatures at higher altitudes may slow down organism matter decomposition, leading to the production of fewer organic acids. Additionally, citrus plants in high-altitude regions face harsher environmental conditions and may develop greater acid tolerance, allowing them to retain more alkaline substances in the soil, thereby reducing soil pH. This observation is consistent with findings from a study conducted in Gorkha, Nepal [72]. At mid-altitude, soil pH values across different land-use types were generally lower than those at high-altitude regions. This is probably because soils tend to contain more hydrogen ions, as higher temperatures at mid-altitude accelerate the decomposition of soil organic matter [73]. Furthermore, the production of NO_3_⁻ ions by plants, combined with the use of acidic fertilizers, may lead to soil acidification [74,75]. Overall, the lower soil pH in mid- to low-altitude regions may be attributed to the decomposition of organic acids from vegetation, the acidifying effects of agricultural management practices, and increased microbial activities. Similar to findings from a study conducted in the mountainous regions of southwest Yunnan Province, China [76], our study also observed an increase in SOC content in CO and SL with altitude (Figure 2). This is probably because as altitude increases, lower soil temperatures lead to reduced microbial activity [51]. Since microbial activity is a key driver of soil organic carbon decomposition [77], its reduction slows the breakdown of organic carbon, promoting the accumulation of organic carbon.

Changes in altitude can directly affect soil physicochemical properties and structure, resulting in significant differences in the composition and diversity of fungal communities across different altitudes. From low to high altitudes, climatic changes lead to significant variations in soil pH. Unlike land use, altitude has a significant effect on SWC, a key environmental factor influencing fungal community composition at lower altitudes (Figure 7). SWC is an important driver of fungal community structure consistent with the results recently reported by Fu et al. [78]. At mid-altitudes, pH is a major factor influencing fungal community composition, as observed in many other studies [34,79,80]. However, some researchers have suggested that fungal abundance may not be strongly affected by pH, or that fungal diversity shows only a weak correlation with pH, possibly due to fungi’s broad tolerance and adaptation to different pH ranges [81]. Across all altitudes, NH_4_^+^-N consistently emerged as a key environmental factor influencing fungal community composition (Figure 7), indicating that soil nutrients have a significant impact on fungal community structure [82,83]. The dominant fungal phyla in our study are Ascomycota and Basidiomycota (Figure 3), which is consistent with results from studies conducted in Changbai Mountain, China [42], Schrankogel Mountain in the Alps [84], and Wuyi Mountain, China [85]. Both phyla have a positive effect on the decomposition of plant residues. Basidiomycetes are generally considered to be more active in the degradation of lignin, while Ascomycetes are mainly responsible for the degradation of plant cell wall polysaccharides (cellulose and hemicellulose, pectin) [86]. Interestingly, the relative abundance of Ascomycota decreased with increasing altitude in both SL and CO. This phenomenon may suggest that ecosystems in high-altitude environments have a reduced ability to decompose plant residues. As the abundance of Ascomycota decreases, the efficiency of soil nutrient cycling could be compromised, negatively affecting the overall health and stability of the ecosystem. This finding also highlights the responsiveness of microbial communities in shrublands and citrus orchards to environmental changes in high-altitude areas, as well as their potential functional roles within these ecosystems. In addition, fungal community composition is influenced by multiple factors, including vegetation type, soil physicochemical properties, and microclimate along altitude gradients [87]. The Ascomycota and Basidiomycota phyla, which are involved in soil organic metabolism [88], affect soil organic matter content through the decomposition of organic matter, thereby determining their abundance. In addition, as a representative of natural vegetation, shrubland, compared to managed land-use types such as CP and CF, lacks human-induced tillage practices, leading to a more compact soil structure with relatively lower porosity and aeration. Reduced aeration limits the diffusion of oxygen and water in the soil, which can hinder the metabolic activity of aerobic fungi [89], thereby affecting the growth and relative abundance of Ascomycota. However, in CP, the relative abundance of Ascomycota increased with altitude. This difference in abundance may be attributed to variations in root exudates from different vegetation types, which can significantly affect fungal community composition [90], suggesting that the responses of fungal communities to altitude vary depending on land-use types [44]. However, the impact of altitude on fungal diversity does not mirror its impact on community composition. The influence of altitude on fungal diversity is shaped by various geographical, climatic, and soil factors [91], making its impact on diversity more complex than on community composition. In our study, NH_4_^+^-N and NO_3_^−^-N were the main environmental factors affecting fungal diversity across different altitudes, consistent with findings from a study conducted in Changbai Mountain, China [42]. Consistent with other research [92], the diversity of SL and CP peaked at 1500 m, with richness indices (Chao1 and ACE) showing an initial decline followed by an increase with altitude. Similarly, the richness indices of CF and CO exhibited a pattern of increasing and then decreasing with altitude, in line with findings from other studies [93]. The Shannon index of SL followed a U-shaped pattern with altitude, while it increased monotonically for the other three land-use types. These findings underscore the complex biogeographical patterns of fungal diversity [94]. We also found that Saprotroph was the dominant fungal functional guild across all altitudes, a common characteristic of fungal communities in many mountain ecosystems [95]. In addition, the relative abundance of Saprotroph increases with altitude, which is consistent with the results recently reported by Guo et al. [96]. This phenomenon may be influenced by multiple factors. In high-altitude areas, lower temperatures slow down the decomposition rate of organic matter, leading to the accumulation of organic matter in the soil [97], which provides abundant substrate sources for saprotroph. In addition, low temperatures and other habitat limitations in high-altitude environments may inhibit the activity of other microbial communities [98], reducing their competition with saprotroph. Under such an environment, saprotroph face lower competitive pressure, and exhibit higher relative abundance by effectively utilizing available resources. Among functional fungi, undefined Saprotroph had the highest relative abundance (Figure 6b) and were positively correlated with pH, but negatively correlated with NH_4_^+^-N, TC, TN, SOC, and DOC. This is consistent with previous studies showing that the composition and abundance of saprotrophic fungi are primarily determined by soil properties [99]. Furthermore, the abundance of arbuscular mycorrhizal fungi (AMF) was influenced by pH and TP. Other studies have identified pH, water content (WC), organic matter (OM) [100], available phosphorus (AP), and nitrogen content as key factors influencing AMF abundance [101]. The variation in AMF abundance with altitude is habitat-specific, as factors such as temperature, precipitation, and frost affect AMF abundance and richness in different ways [102]. In our study, functional fungi were strongly influenced by soil physicochemical properties. Notably, the correlation between pH and functional fungi diminished with increasing altitude, as functional fungi exhibit habitat-specific adaptations [103]. For instance, AMF prefer environments with near-neutral soil pH [104], ectomycorrhizal fungi (EMF) are more suited to acidic soils [105], while pathogenic fungi are less influenced by pH and more by TN content [106].

### 4.3. Comparative and Combined Effects of Land Use and Altitude on Fungal Communities

In our study, the combined effects of land use and altitude significantly influenced fungal community structure. However, fungal communities’ responses to land use and altitude varied. Compared to land-use types, the alpha diversity and functional guilds of fungal communities were more sensitive to changes in altitude. Specifically, altitude had a significant impact on the Chao1 index, ACE index, Shannon index, and Simpson index of fungal communities. Additionally, functional fungi with a relative abundance level greater than 1% were significantly impacted by altitude (Table 2, *p* < 0.001). In contrast, the Simpson index of fungal communities and certain functional fungi showed no significant correlation with land-use types (*p* > 0.05). In mountain ecosystems, altitude is the primary factor influencing fungal microbial communities [107]. Altitude affects plants, soil, and climate, which in turn strongly shape microbial community diversity [93]. Environmental variation due to altitude is more persistent and widespread, primarily reflected in changes in soil properties and geographic distance, which directly affect fungal community diversity and functional guilds. Studies have shown that soil pH is the main factor influencing fungal alpha diversity in response to altitude changes, while beta diversity and turnover of total fungi and saprotrophic fungi are shaped by soil properties and geographic distance [34]. In contrast, the influence of land use tends to be more localized, indirectly affecting fungal community composition by altering soil physicochemical properties. For example, studies have indicated that soil phosphorus can significantly affect the abundance of Ascomycota, suggesting that land use affects fungal communities by altering soil properties [108]. Additionally, altitude-driven effects are typically shaped by natural factors, whereas land-use impacts are more subject to human interventions.

Land use has a greater impact on fungal community composition than altitude, as it significantly affects most fungal phyla, with the exception of Blastocladiomycota. The PCoA analysis further supports this, showing clear separation distances between different land-use types at the same altitude, indicating that land-use types have significantly altered fungal community composition. In contrast, except for CP, there was no substantial shift in distance for the same land-use type across different altitudes, suggesting that altitude has a relatively smaller effect on fungal community composition. Similarly, a study examining land use and spatial distances between land uses found that different land-use types at the same location were distinctly separated in NMDS analyses, indicating that land use has a more profound and evident effect on fungal community composition [109]. Another study also observed that land-use types strongly influenced fungi community composition in tropical regions of China [110]. A recent study examining three influencing factors—land-use intensity, grazing, and fire disturbance—concluded that their interaction affects microbial community structure, with fungal communities showing greater sensitivity to land-use change [111]. Overall, land-use change is one of the most critical environmental factors affecting soil characteristics, microbial community structure, and soil ecological functions [112,113]. Notably, pronounced changes in fungal community composition due to land-use practices are frequently reported [114]. Lastly, the interaction between land use and altitude significantly affects soil properties, fungal diversity, community composition (excluding Blastocladiomycota), and functional guilds. The relative abundance of functional fungi, in particular, was significantly influenced by the interaction between land use and altitude.

## 5. Conclusions

In summary, the present study highlights the impact of land use and altitude on soil fungal community composition and diversity in the Gaoligong Mountain region. Significant differences were observed in soil physicochemical properties and fungal community structures across various land-use types and altitudes. First, fungal community alpha diversity and functional guilds were more sensitive to altitude changes, whereas fungal community composition was more strongly influenced by land-use changes. Additionally, the interaction between land use and altitude had a significant impact on soil fungal community structure. The dominant fungal phyla are Ascomycota and Basidiomycota, while the dominant genera are *Fusarium* and *Mortierella*. Key factors influencing the composition of fungal communities at different altitudes included soil pH, NH_4_^+^-N, SWC, and NO_3_^−^-N. Among these, soil NH_4_^+^-N and NO_3_^−^-N were critical in affecting fungal community diversity, whereas SWC showed no significant correlation with most fungal functional guilds. We concluded that land use and altitude had a significant impact on the structure and diversity of soil fungal communities in mountain ecosystems. Understanding the integrated effects of these two factors can help us gain a more comprehensive insight into the spatial and temporal dynamics of soil fungal communities and their ecological functions under changing environmental conditions.

## Figures and Tables

**Table 1:** The results of two-way analysis of variance analyzing the effects of land use, elevation, and their interactions on the relative abundance of different fungal phyla in soil samples from the Gaoligong Mountain region.

Phylum	Land Use		Altitude		Interaction	
	F	*p*	F	*p*	F	*p*
Ascomycota	12.213	<0.001	126.379	<0.001	17.806	<0.001
Basidiomycota	27.841	<0.001	5.759	<0.01	4.961	<0.01
Mortierellomycota	8.194	<0.001	33.937	<0.001	95.155	<0.001
Unassigned	13.482	<0.001	10.52	<0.001	6.599	<0.001
Chytridiomycota	5.778	<0.01	5.383	<0.05	2.882	<0.05
Rozellomycota	35.561	<0.001	16.544	<0.001	3.737	<0.01
Glomeromycota	37.588	<0.001	7.642	<0.01	23.595	<0.001
Olpidiomycota	10.397	<0.001	10.532	<0.001	10.429	<0.001
Kickxellomycota	30.606	<0.001	18.797	<0.001	13.366	<0.001
Blastocladiomycota	0.411	0.747	1.229	0.31	1.252	0.316

**Table 2:** The results of two-way analysis of variance analyzing the effects of land use, elevation, and their interactions on the relative abundance of different guilds of fungi in soil samples from the Gaoligong Mountain region.

Guild	Land Use		Altitude		Interaction	
	F	*p*	F	*p*	F	*p*
Undefined Saprotroph	0.764	0.526	13.056	<0.001	8.925	<0.001
Epiphyte	13.459	<0.001	22.846	<0.001	14.687	<0.001
Endophyte-Litter Saprotroph-Soil Saprotroph-Undefined Saprotroph	9.8	<0.001	13.362	<0.001	92.557	<0.001
Arbuscular Mycorrhizal	40.282	<0.001	13.522	<0.001	25.556	<0.001
Wood Saprotroph	19.061	<0.001	43.719	<0.001	19.474	<0.001
Animal Pathogen-Fungal Parasite-Undefined Saprotroph	10.694	<0.001	28.859	<0.001	15.26	<0.001
Animal Pathogen-Undefined Saprotroph	12.714	<0.001	10.557	<0.001	6.31	<0.001
Plant Pathogen-Wood Saprotroph	20.534	<0.001	24.387	<0.001	36.878	<0.001
Plant Pathogen-Undefined Saprotroph	4.902	<0.01	10.756	<0.001	5.418	<0.01
Endophyte-Lichen Parasite-Plant Pathogen-Undefined Saprotroph	4.331	<0.05	36.939	<0.001	12.999	<0.001

**Table 3:** Correlation of fungal community diversity with physicochemical properties.

Altitude		pH	TC	TN (g·kg^−1^)	TP (mg·kg^−1^)	NH_4_^+^-N (mg·kg^−1^)	NO_3_^−^-N (mg·kg^−1^)	SOC (g·kg^−1^)	DOC (g·kg^−1^)	SWC (%)
900 m	Chao1	0.297	−0.355	−0.423	−0.394	0.735 **	−0.862 **	−0.361	−0.449	−0.035
	ACE	0.319	−0.362	−0.423	−0.401	0.735 **	−0.855 **	−0.361	−0.438	−0.021
	Shannon	0.107	−0.313	−0.38	−0.418	0.271	−0.371	−0.406	−0.537	−0.119
	Simpson	−0.2	0.035	0.077	0.049	−0.018	0.144	0.112	0.251	0.126
1200 m	Chao1	−0.013	0.614	0.555	−0.268	−0.917 **	0.915 **	0.569	0.012	0.195
	ACE	−0.013	0.614	0.555	−0.268	−0.917 **	0.915 **	0.569	0.012	0.195
	Shannon	0.151	0.711 *	0.713 *	−0.591	−0.789 **	0.685 *	0.654 *	0.244	−0.322
	Simpson	−0.238	−0.729 *	−0.738 *	0.744 *	0.661 *	−0.515	−0.691 *	−0.36	0.468
1500 m	Chao1	−0.810 **	0.364	0.702 *	0.141	0.667 *	0.713 **	0.772 **	0.716 **	0.51
	ACE	−0.810 **	0.364	0.702 *	0.141	0.667 *	0.713 **	0.772 **	0.716 **	0.51
	Shannon	−0.789 **	0.294	0.505	0.088	0.642 *	0.713 **	0.621 *	0.688 *	0.336
	Simpson	0.106	−0.287	0.091	−0.063	0.211	−0.028	−0.007	−0.099	0.175

* *p* < 0.05, ** *p* < 0.01.

## Data Availability

The original contributions presented in the study are included in the article/Supplementary Material, further inquiries can be directed to the corresponding author.

## Supplementary Materials

The following supporting information can be downloaded at: https://www.mdpi.com/article/10.3390/microorganisms12112378/s1, Figure S1: The dilution curves of soil fungal amplicon sequence variants (ASVs) for four different land-use communities at different altitudes. ((**a**–**c**) correspond to 900 m, 1200 m and 1500 m).
