# Supplementary material for: Elevation Determines Fungal Diversity, and Land Use Governs Community Composition: A Dual Perspective from Gaoligong Mountains
Source: Microorganisms. 2024 Nov 20;12(11):2378. doi: 10.3390/microorganisms12112378 (PMC11596228; doi:10.3390/microorganisms12112378)
Supplement: Supplementary file 1 [file microorganisms-12-02378-s001.zip › microorganisms-3242467-supplementary.pdf]

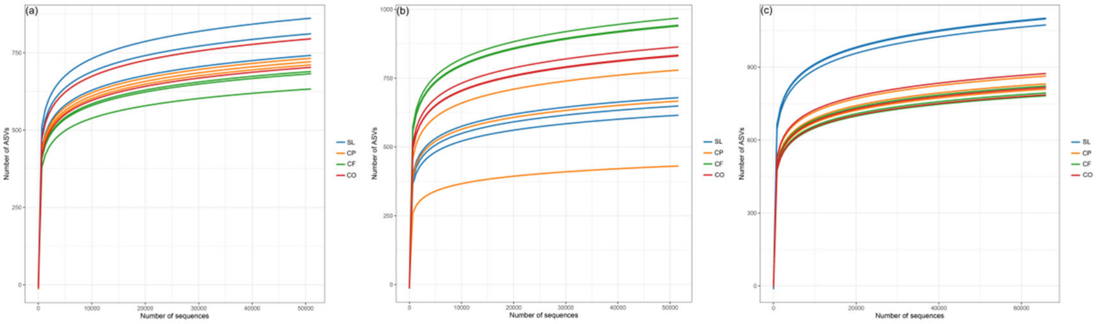

Figure S1. The dilution curves of soil fungal amplicon sequence variants (ASVs) for four different land use communities at different altitudes. ((**a**–**c**) correspond to 900m, 1200m and 1500m).
